# Biocompatibility, Surface Morphology, and Bacterial Load of Dental Implant Abutments following Decontamination Protocols: An In-Vitro Study

**DOI:** 10.3390/ma16114080

**Published:** 2023-05-30

**Authors:** Esi Sharon, Yoav Pietrokovski, Ilana Engel, Rula Assali, Yael Houri-Haddad, Nurit Beyth

**Affiliations:** Department of Prosthodontics, Hadassah Medical Center, Faculty of Dental Medicine, Hebrew University of Jerusalem, Jerusalem 9112102, Israel; esisharon@gmail.com (E.S.); ilana.engel@mail.huji.ac.il (I.E.); rulaassali@gmail.com (R.A.); nurit.beyth@mail.huji.ac.il (N.B.)

**Keywords:** peri-implantitis, titanium, decontamination, steam, ultrasonics, osseointegration

## Abstract

The long-term success of dental implant rehabilitation depends significantly on proper peri-implant soft tissue integration. Therefore, decontamination of abutments prior to their connection to the implant is beneficial to enhance soft tissue attachment and to aid in maintaining marginal bone around the implant. Consequently, different implant abutment decontamination protocols were evaluated regarding biocompatibility, surface morphology, and bacterial load. The protocols evaluated were autoclave sterilization, ultrasonic washing, steam cleaning, chlorhexidine chemical decontamination, and sodium hypochlorite chemical decontamination. The control groups included: (1) implant abutments prepared and polished in a dental lab without decontamination and (2) unprepared implant abutments obtained directly from the company. Surface analysis was performed using scanning electron microscopy (SEM). Biocompatibility was evaluated using XTT cell viability and proliferation assays. Biofilm biomass and viable counts (CFU/mL) (n = 5 for each test) were used for surface bacterial load evaluation. Surface analysis revealed areas of debris and accumulation of materials, such as iron, cobalt, chromium, and other metals, in all abutments prepared by the lab and with all decontamination protocols. Steam cleaning was the most efficient method for reducing contamination. Chlorhexidine and sodium hypochlorite left residual materials on the abutments. XTT results showed that the chlorhexidine group (M = 0.7005, SD = 0.2995) had the lowest values (*p* < 0.001) (autoclave: M = 3.6354, SD = 0.1510; ultrasonic: M = 3.4077, SD = 0.3730; steam: M = 3.2903, SD = 0.2172; NaOCl: M = 3.5377, SD = 0.0927; prep non-decont.: M = 3.4815, SD = 0.2326; factory: M = 3.6173, SD = 0.0392). Bacterial growth (CFU/mL) was high in the abutments treated with steam cleaning and ultrasonic bath: 2.93 × 10^9^, SD = 1.68 × 10^12^ and 1.83 × 10^9^, SD = 3.95 × 10^10^, respectively. Abutments treated with chlorhexidine showed higher toxicity to cells, while all other samples showed similar effects to the control. In conclusion, steam cleaning seemed to be the most efficient method for reducing debris and metallic contamination. Bacterial load can be reduced using autoclaving, chlorhexidine, and NaOCl.

## 1. Introduction

Implant-supported single crowns and fixed prosthetic dentures are considered successful treatment modalities for the replacement of lost teeth [1,2]. The 5-year survival rate of implants supporting single crowns was reported to be 96.8%, and the survival rate of single crowns supported by implants was reported to be 94.5% [3]. The 10-year survival rate of implants supporting single crowns was 95.2%, and the survival rate of single crowns supported by implants was 89.4% [4]. There is considerable evidence to support the view that the long-term success of implant rehabilitation depends significantly on several factors, including proper peri-implant soft tissue integration, which serves as a protective barrier between the oral environment and the underlying peri-implant bone [5,6]. The peri-implant mucosa is generally recognized as hypovascular and hypocellular scar tissue; due to the lack of a periodontal ligament, the only source of nourishment comes from the supraperiosteal blood vessels [7,8]. It is immunologically highly inferior to the periodontal tissues around teeth, since it exhibits impaired resistance to bacterial colonization [9,10] and, in turn, renders dental implants more susceptible to inflammation and subsequent bone loss from microbial challenges [7,8].

Peri-implantitis and soft tissue complications occur adjacent to 9.7% of single crowns, and 6.3% of implants show bone loss exceeding 2 mm over a 5-year observation period [3]; therefore, in addition to the thickness of the mucosa, adequate soft tissue integration at the transmucosal part of the implant is important in order to seal the adjacent alveolar bone from the oral environment [11,12,13].

The most widely used abutment material is titanium, as it has proven long-term clinical success [14]. It has good biocompatibility, along with high resistance to corrosion [15]. Some claim that there may be cases in which stock abutments, either for cemented- or screw-retained restorations, are not adjusted and polished [16]. However, in most cases, as part of the abutment preparation, whether it is CAD/CAM or custom, they are adjusted and polished by a technician. This process can possibly lead to contamination of the abutment surface with debris and other contaminants. Furthermore, CAD/CAM abutments with no manual lab adjustments can potentially be contaminated by the milling process itself. This contamination can include various microorganisms and debris. Microorganisms and their endotoxins can cause osteoclastogenesis and subsequent bone resorption [17]. The debris, which may include titanium microparticles produced during abutment customization, can act as a foreign body and produce inflammatory responses that may influence soft tissue healing around implants [18].

Plaque formation and bacterial colonization influenced by the surface properties of the abutment and implant collar are considered to play key roles in the pathogenesis of infections [19,20]. At the same time, microbiological contamination due to different laboratory steps and auxiliary staff management has been documented on customized prosthetic components [21].

Contamination can affect the aforementioned adhesion of soft tissue to the dental abutment surface, which is required for the long-term success of the restoration, as it establishes a biological seal between the soft tissue and the implant surface, limiting bacterial penetration, gingival recession, and bone recession [22]. Other potential clinical implications of bacterial invasion of the implant may be associated with tribocorrosion, a chemo-mechanical degradation process in which particles from titanium surfaces are released in the oral environment, which increases tissue inflammation [23], thus emphasizing the need and importance of a reliable and practical decontamination protocol that can be adapted to everyday clinical practice [24,25].

It is therefore advised to decontaminate abutments sent from dental laboratories prior to connecting them with implants in order to enhance soft tissue attachment and aid in maintaining marginal bone around implants [26]. Various groups have tested different decontamination and sterilization methods on implant abutments, including steam cleaning [27], autoclave sterilization [28], sodium hypochlorite chemical decontamination [29], ultrasonic washing [30], use of plasma of argon [24], and others [31], with varying rates of success.

The study hypothesis was that different decontamination methods would not affect the abutment’s biocompatibility, would not cause any changes to its surface morphology, and would not affect the resultant bacterial load. The aim of this study was to evaluate and compare the effects of different decontamination protocols on both the abutment’s biocompatibility and its surface morphology.

## 2. Materials and Methods

### 2.1. Titanium Abutment Preparation

A total of 105 custom-made titanium standard cementing implant posts (MD-MAC10, M.I.S Implants Technologies Ltd., Misgav, Israel) were used in the study (Ti-6 Al-4V Grade 23, according to manufacturer data sheet information).

Sixty custom-made titanium implant abutments were prepared and polished in a dental lab using a round-end taper tungsten carbide bur used on titanium (FG.TC085618 bur, M.D.T Micro Diamond Technologies Ltd., Afula, Israel; head diameter: 1.8 mm, head length: 8 mm). The preparation parameters were standardized and performed similarly on all abutments. These parameters included axial and occlusal reduction of the abutments, with a standard chamfer finishing line.

Following this step, five decontamination protocols were performed: autoclave sterilization, ultrasonic washing, steam cleaning, chlorhexidine chemical decontamination, and sodium hypochlorite chemical decontamination. Each group of abutments was treated with one protocol only (except for the control groups, which were not treated at all).

Autoclave sterilization was performed at 121 °C for 50 min, ultrasonic washing was performed by placing the abutments in an ultrasonic bath for 15 min, steam cleaning was performed by steaming the abutments for 5 s in 4 MPa pressure, chlorhexidine chemical decontamination was performed by placing the abutments in a 2% chlorhexidine gluconate solution for 10 min, and sodium hypochlorite chemical decontamination was performed by immersing the abutments in a 2.5% NaOCl solution for 10 min. Each decontamination method was performed on 10 abutments, so there were 5 abutments for each of the experiments (SEM analysis and XTT biocompatibility were tested on the same abutments).

1. Surface analysis using scanning electron microscopy (SEM).
2. XTT assay for cell viability and proliferation.
3. Bacterial direct count of colony-forming units per milliliter (CFU/mL).

The control groups included: (1) implant abutments prepared and polished in a dental lab without decontamination follow-up (n = 10; 5 for each experiment) and (2) implant abutments tested directly from the factory that were not prepared in a dental lab (n = 6; 5 for the biocompatibility experiment and 1 for surface analysis).

### 2.2. Surface Analysis by SEM

Surface analysis was performed using SEM analysis [32]. SEM was performed with a VEGA3 (TESCAN ANALYTICS, Brno, Czech Republic) scanning electron microscope (SEM) at 20 kV in secondary electrons (SE) and backscattered electrons (BSE) mode; this analysis was performed on several samples: 2 abutments from the factory that did not undergo any lab preparation, 2 abutments that were prepared in a lab but did not undergo any decontamination method, 2 abutments that were decontaminated with steam cleaning, 2 abutments that were decontaminated with ultrasonic washing, and 1 abutment for each of the other decontamination groups (autoclave, chlorhexidine, and sodium hypochlorite). The magnifications in which the abutments were scanned and compared were ×60, ×500, ×1000, and ×2000.

Chemical element analysis of the samples was performed using a Bruker EDX (Bruker, Bremen, Germany) apparatus on the SEM.

### 2.3. XTT Assay for Cell Viability and Proliferation

The biocompatibility experiment was performed using XTT assay [33,34]. Five abutments for each decontamination group (autoclave, ultrasonic washing, steam cleaning, chlorhexidine, and sodium hypochlorite), 5 abutments that were prepared in a lab but did not undergo any decontamination method, and 5 abutments from the factory that were not prepared in a lab were tested.

The cells that were used were RAW 264.7 (ATCC TIB-71, Manassas, VA, USA) macrophage cell line, cultured in Petri dishes containing Dulbecco’s Minimum Essential Medium (DMEM) supplemented with 10% fetal calf serum (FCS), 1% penicillin/streptomycin, and 1% glutamine. The cells were seeded at a density of 60,000 cells/well in a 96-well tissue culture plate (NUNC). Various abutments were added into the wells; 24 h after plating, the cells were activated with heat-killed porphyromonas gingivalis 33277 ATCC and incubated for 24 h at 37 °C in a humidified atmosphere of 5% CO_2_. Cell viability was evaluated using a colorimetric XTT assay, as described by Scudiero et al. [35], based on the metabolic reduction of soluble tetrazolium salts (XTT) to insoluble orange-colored formazans. Fifty milliliters of XTT labeling mixture was added to each well, and the microplates were incubated for a further 4 h. A Vmax microplate reader (Molecular Devices Corporation) with a 450 nm optical filter and a 650 nm reference wavelength was used to measure the absorbance of each well.

### 2.4. Bacterial Evaluation

Forty-five custom-made titanium implant abutments were randomly divided into nine groups of five samples each. Forty implant abutments were prepared and polished, as described above, by a dental laboratory technician. The control groups included two groups of 5 abutments each: one group included implant abutments tested directly from the factory, and the other group included implant abutments prepared and polished in a dental lab without further treatment.

All groups were contaminated overnight with Enterococcus faecalis bacteria. E. faecalis bacteria was grown for 24 h at 37 °C under aerobic conditions in brain heart infusion (BHI) medium (Difco Laboratories, Detroit, MI, USA). Cultures were diluted 1:1000 into a new tube with fresh BHI medium and grown for 2 h to the mid-log phase, and optical density was adjusted to OD650 nm = 0.1 for the experiments. A viable count (CFU/mL) was performed for bacterial contamination evaluation.

Abutment decontamination was performed as described above. The control groups were: (1) adjusted titanium abutments without further treatment and (2) unadjusted titanium abutments. Abutments were first contaminated for 24 h, and the baseline in all groups following contamination was 10^9^ CFU/mL. Then, the abutments were subjected to decontamination protocols. After decontamination, each abutment was placed in BHI broth for 24 h in a 96-well microtiter plate at 37 °C, and viable counts (CFU/mL) were evaluated.

### 2.5. Statistical Analysis

Statistical analysis was performed using a one-way ANOVA test that compared the various decontamination groups using the F distribution.

Mean values and standard deviations were calculated for the control and test groups. All comparisons were performed using the ANOVA test with the post hoc Tukey multiple comparison test. The level of statistical significance was set at *p* ≤ 0.05. Statistical analysis was performed using IBM SPSS Statistics v. 20.0 software (Chicago, IL, USA).

## 3. Results

### 3.1. Surface Analysis by Scanning Electron Microscopy (SEM)

**Figure 1:**
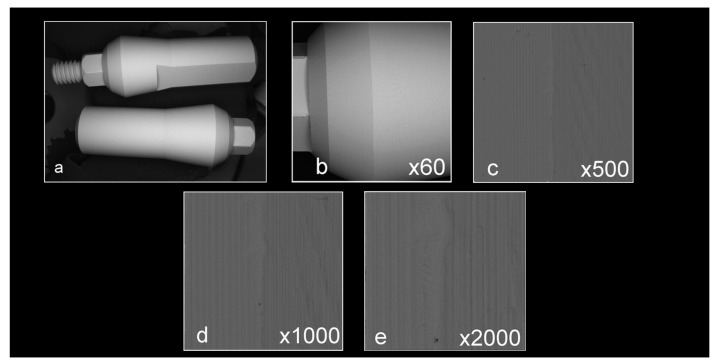
Factory abutments that did not undergo laboratory preparation ((**a**) no magnification; (**b**) ×60 magnification; (**c**) ×500 magnification; (**d**) ×1000 magnification; (**e**) ×2000 magnification). The samples are clean, smooth, and devoid of any signs of debris and/or contamination.

**Figure 2:**
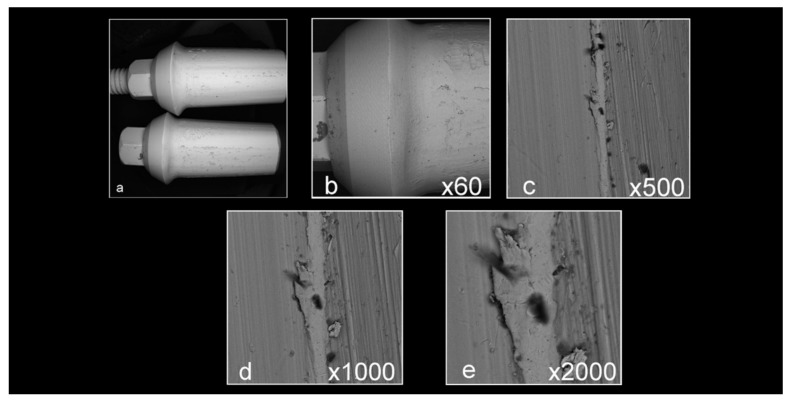
Abutments that were prepared in a lab but did not undergo any decontamination method ((**a**) no magnification; (**b**) ×60 magnification; (**c**) ×500 magnification; (**d**) ×1000 magnification; (**e**) ×2000 magnification). Apart from the noticeable scratches caused by the tungsten carbide burs used to prepare the abutments, the samples are covered with various debris and contaminants.

**Figure 3:**
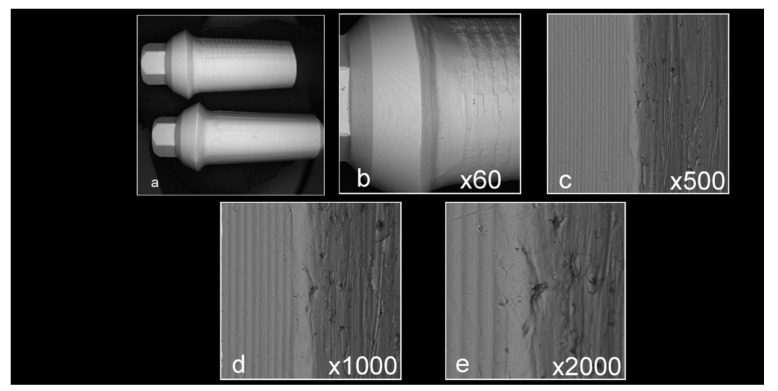
Abutments that were prepared in a lab and decontaminated using steam cleaning ((**a**) no magnification; (**b**) ×60 magnification; (**c**) ×500 magnification; (**d**) ×1000 magnification; (**e**) ×2000 magnification). The samples have some debris, but to a lesser extent than the control samples in Figure 2 and look smoother.

**Figure 4:**
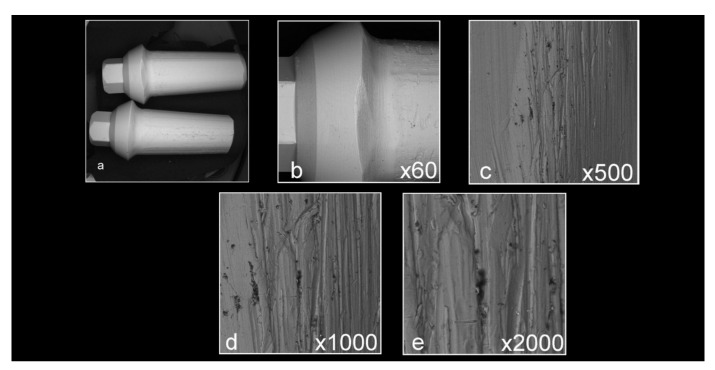
Abutments that were prepared in a lab and decontaminated using ultrasonic washing ((**a**) no magnification; (**b**) ×60 magnification; (**c**) ×500 magnification; (**d**) ×1000 magnification; (**e**) ×2000 magnification). The samples have some debris and accumulation of contaminants, but look smoother compared to the control samples shown in Figure 2.

**Figure 5:**
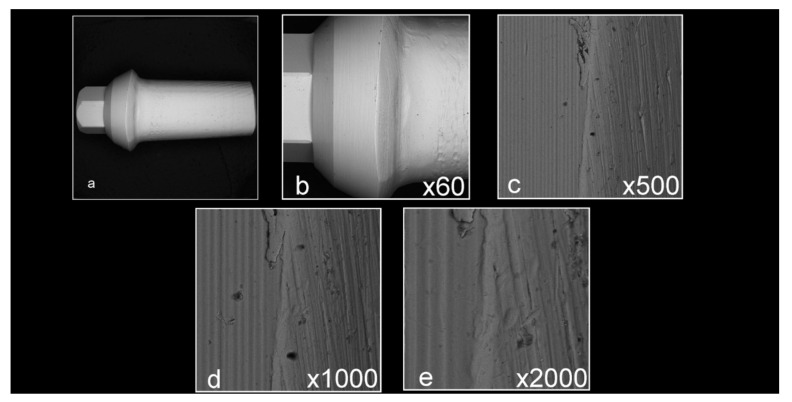
Abutment that was prepared in a lab and decontaminated using autoclave sterilization ((**a**) no magnification; (**b**) ×60 magnification; (**c**) ×500 magnification; (**d**) ×1000 magnification; (**e**) ×2000 magnification). The sample has some debris and accumulation of contaminants, but to a lesser extent than the control samples in Figure 2.

**Figure 6:**
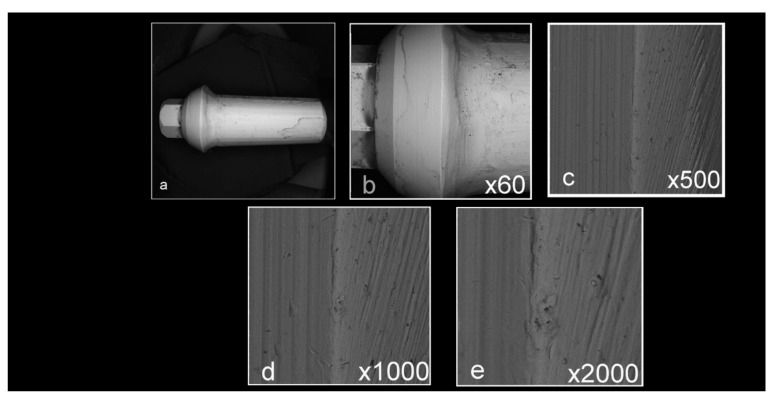
Abutment that was prepared in a lab and decontaminated using chlorhexidine ((**a**) no magnification; (**b**) ×60 magnification; (**c**) ×500 magnification; (**d**) ×1000 magnification; (**e**) ×2000 magnification). The sample has some debris and accumulation of contaminants, but to a lesser extent than the control samples in Figure 2. In addition, the sample is covered with residual material from the CHX solution.

**Figure 7:**
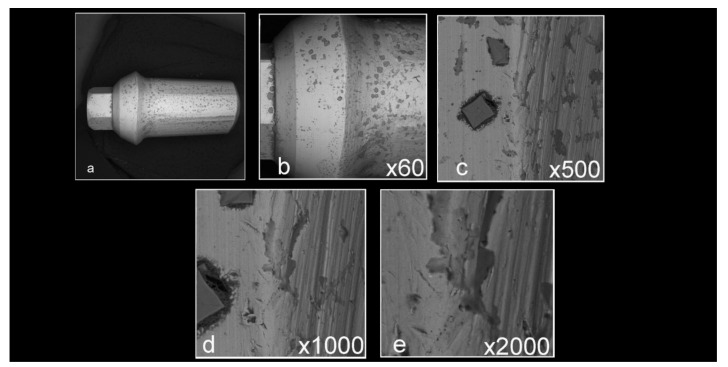
Abutment that was prepared in a lab and decontaminated using sodium hypochlorite ((**a**) no magnification; (**b**) ×60 magnification; (**c**) ×500 magnification; (**d**) ×1000 magnification; (**e**) ×2000 magnification). It is noticeable that, in comparison to the control samples seen in Figure 2, the sample is covered with major residual material from the NaOCl solution, as well as pits and flaws apparently caused by the sodium hypochlorite solution.

**Figure 8:**
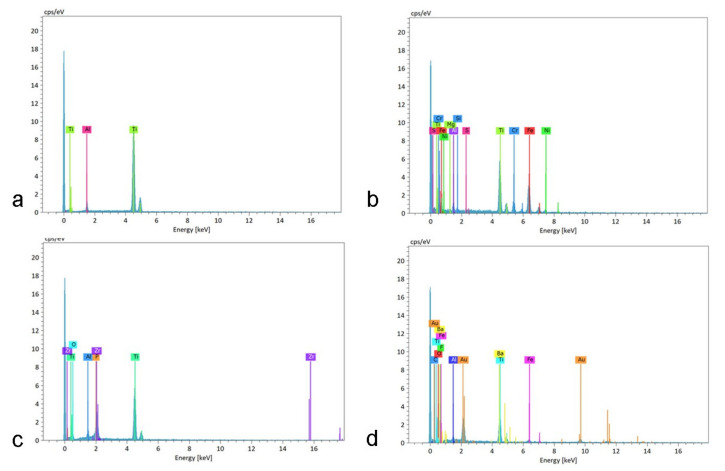
Chemical analysis of abutments under different treatments. The different peaks represent various metallic contaminations that were analyzed on the samples. (**a**) abutments from factory, (**b**) abutments after lab preparation, (**c**) abutments after lab preparation and steam cleaning, and (**d**) abutments after lab preparation and ultrasonic washing.

Abutment surface views following various decontamination protocols, as depicted using SEM, were recorded at increasing magnification: no magnification, ×60, ×500, ×1000, and ×2000 (Figure 1, Figure 2, Figure 3, Figure 4, Figure 5, Figure 6, Figure 7). Five samples per group were tested. Chemical analysis showed pinpoint metallic contaminants, such as iron, cobalt, chromium, nickel, magnesium, palladium, tin, gold, copper, zirconium, and tungsten (Figure 8). Among the different groups, steam cleaning showed the least amount of metals compared to the other groups, although metallic contamination was still found in that group as well. The two decontamination solutions (chlorhexidine and sodium hypochlorite) left residual materials on the abutments. The results are shown in Figure 1, Figure 2, Figure 3, Figure 4, Figure 5, Figure 6, Figure 7, Figure 8.

### 3.2. XTT Assay for Cell Viability and Proliferation

The cytotoxic effect of the various decontamination methods was evaluated using an XTT assay. Five samples per group were tested. One-way ANOVA analysis showed statistically significant differences among the groups (F(6,16) = 78.896, *p* < 0.001). The CHX values were significantly lower than the rest of the groups, which, in turn, showed similar values to each other.

**Figure 9:**
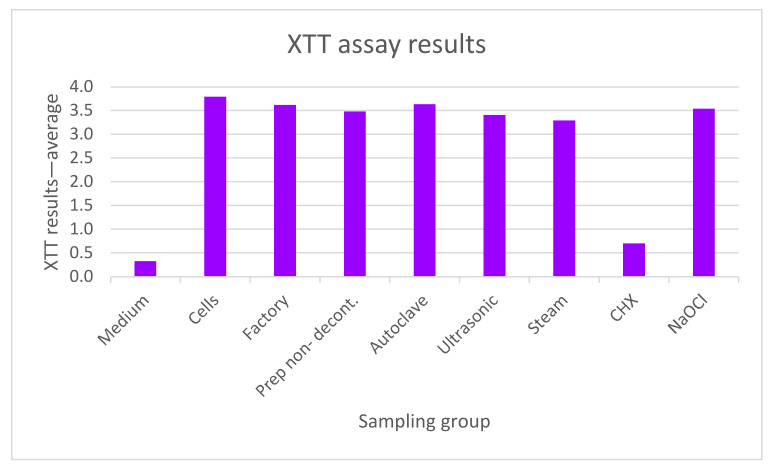
XTT assay results. The various decontamination methods were assessed (autoclave, ultrasonic washing, steam cleaning (steam), chlorhexidine (CHX), and sodium hypochlorite (NaOCl)), as well as 5 abutments that were prepared in a lab but did not undergo any decontamination method (prep non-decont.) and 5 abutments from the factory that did not undergo any decontamination method (factory). Additional control groups included macrophage-like raw cells cultured in medium (cells), as well as supplemented with DMEM (medium). The average XTT values of CHX were significantly lower than the XTT values of any of the other groups and close to the values of the supplemental DMEM (medium). The rest of the groups showed XTT values similar to each another.

The results are shown in Figure 9 and Table 1.

Further analysis (Scheffe’s test) showed that the XTT values of the CHX group (M = 0.7005, SD = 0.2995) were significantly—almost five times—lower (*p* < 0.001) than the XTT values of the other groups (autoclave: M = 3.6354, SD = 0.1510; ultrasonic: M = 3.4077, SD = 0.3730; steam: M = 3.2903, SD = 0.2172; NaOCl: M = 3.5377, SD = 0.0927; prep non-decont: M = 3.4815, SD = 0.2326; factory: M = 3.6173, SD = 0.0392).

Further statistical analysis was completed to confirm the statistical difference between the CHX group and the other groups. The CHX values were almost five times lower than the rest of the groups. The other groups showed similar values. The results are shown in Table 2.

### 3.3. Bacterial Evaluation

**Figure 10:**
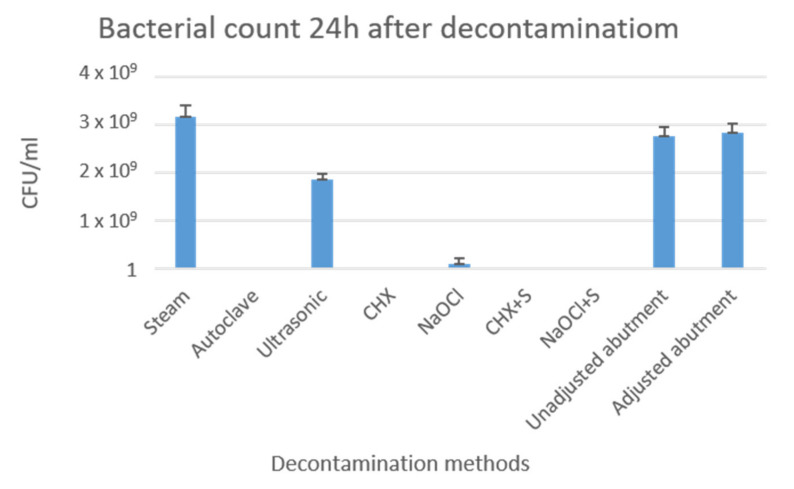
Comparison of mean bacterial counts (CFU/mL) following decontamination procedures. Groups 1 and 2 (controls: unprepared untreated abutments and prepared untreated abutments). Groups 3–9 abutments after different decontamination procedures. No bacterial growth was observed in the implant abutments treated with autoclave, chlorhexidine, chlorhexidine + saline, NaOCl solution, and NaOCl solution + saline. Very low bacterial growth was seen in implant abutments treated with chlorhexidine. Abutments treated with steam cleaning and ultrasonic bath showed bacterial growth closely resembling the control groups.

Five samples per group were tested. As seen in Table 3 and Figure 10, no bacterial growth was observed in the implant abutments treated with autoclave, chlorhexidine, chlorhexidine + saline, and NaOCl solution + saline. Very low counts were seen in the NaOCl solution group. However, in the abutments treated with steam cleaning and ultrasonic bath, bacterial growth was observed. The average number of colony forming units (CFU/mL) was 3.18 × 10^9^ with SD: 2.36 × 10^8^ and 1.85 × 10^9^ with SD: 1.29 × 10^8^, respectively (Table 3, Figure 10).

The mean bacterial count (CFU/mL) following the decontamination procedures of the tested samples (ANOVA) showed the highest mean bacterial count in the ultrasonic bath treatment and the steam cleaning group. Tukey multiple comparison tests highlighted significant differences between these two groups and the other groups (*p* < 0.05) (Table 3, Figure 10).

## 4. Discussion

The survival of dental implants is affected by the quality of the mucosal attachment, and the abutment properties directly affect it. The process that the abutment goes through from the moment it leaves the factory and from the technician to the physician is not sterile; therefore, appropriate decontamination must be performed before inserting it into the patient’s mouth. The aim of this study was to investigate different decontamination protocols on titanium implant abutments after laboratory preparation and before clinical application in order to suggest a reliable and practical decontamination method for use in clinical practice. Surface analysis of the abutments that underwent the lab preparation procedure revealed areas of debris and accumulation of materials that were analyzed chemically and found to contain various metallic contaminants. This occurred in all the abutments that were prepared by the lab and under all decontamination methods. Steam cleaning seemed to be the most efficient method for reducing contamination, although abutments that underwent steam cleaning were still found to have metallic contamination. From a clinical point of view, it is vital to ensure that abutments prepared by a dental lab, which are to be tried in and delivered to patients’ mouths, undergo a useful, practical, and reliable decontamination method in order to promote adequate soft tissue adhesion, which is crucial for a biological seal between the implant surface and the soft tissue. This, in turn, can limit bacterial penetration and help prevent the unfortunate cascade of events that are seen more and more these days, including gingival and bone recession, as well as peri-implant diseases [13]. All the decontamination methods tested in this experiment are practical and can be used daily in a dental lab or clinic, although none of the various decontamination methods tested showed complete removal of debris. This, in turn, questions their usefulness and reliability and indicates that decontamination methods must be improved.

This leads to the speculation that there is a need to use decontamination combining not only chemical decontamination but mechanical cleaning as well in order to reduce the amount of metallic contamination observed. The two decontamination solutions used in this experiment (chlorhexidine and sodium hypochlorite) left residual materials on the abutments; therefore, in the case of chemical decontamination that involves immersing an abutment in a decontamination medium, it might be beneficial to rinse the abutment in sterile water or saline following the decontamination procedure.

In this study, the surfaces of the tungsten carbide burs that were used to prepare the abutments were investigated as well, and chemical analysis of the various elements found on them was executed. Interestingly, the analysis revealed gold particles originating on the bur’s shank. The gold particles also appeared on the active part of the bur. Titanium particles were observed as well. It can be surmised that the gold contamination on the titanium abutments depicted in this study probably originated from the tungsten bur’s shank. The other metallic contaminants that were found on the abutments probably originated from elsewhere, such as contamination from other dental materials present in the lab. This strengthens the aforementioned speculation that it is beneficial to use a decontamination method that combines mechanical cleaning as well.

Considering that steam cleaning showed the best results, it is recommended to combine mechanical cleaning of abutments with steam cleaning in order to reduce the level of debris and contaminants on the abutment. This is further supported by studies evaluating air polishing of implant surfaces, which have shown efficient surface decontamination [36].

It is important for the decontamination method to be biocompatible and nontoxic to the surrounding environment, as well as to have minimal or no effect on the abutment surface. In this study, the biocompatibility of the different decontamination methods was tested through XTT assay. The viability levels of cells are good indicators of cell health. Physical and chemical agents can affect cell health and metabolism by causing toxicity to cells via different mechanisms (e.g., destruction of cell membranes, prevention of protein synthesis, irreversible binding to receptors, inhibition of polydeoxynucleotide elongation, and enzymatic reactions) [37]. In order to determine the cell death caused by these mechanisms, there is a need for cheap, reliable, and reproducible short-term cytotoxicity and cell viability assays, much like the XTT assay.

This experiment showed that placing the abutments in chlorhexidine resulted in significantly higher toxicity of the abutments to the surrounding cells, while all other samples showed values that were similar to the control. This might be due to residual CHX on the abutments (which was also observed under SEM) that may have been toxic to the surrounding cells. Similar to the speculation in the previous experiment, it might therefore be beneficial to rinse abutments in sterile water or saline following chemical decontamination, which involves putting the abutment in a decontamination medium; interestingly, however, higher toxicity to the surrounding cells was not observed for abutments soaked in sodium hypochlorite. Another decontamination solution that was not tested in this study but is also worth considering is H_2_O_2_, which is showing promising results as far as effective internal decontamination of implants [38].

Regarding the other decontamination methods, it seems they are all biocompatible with their surroundings, with the abutments that were decontaminated in the autoclave showing results that were the closest to the control. With regard to biologic contamination of the implant abutments, the results of the present study show that the degrees of decontamination—the mean bacterial counts—were greatly influenced by different cleaning procedures. The results confirmed that abutments treated with steam cleaning, a widely used method after laboratory work, and ultrasonic bath treatment remained contaminated. The other five decontamination methods, namely autoclaving, chlorhexidine treatment, NaOCl treatment, chlorhexidine treatment followed by saline, and NaOCl treatment followed by saline, significantly reduced contamination, with mean bacterial counts of 0. The abutment surfaces’ bacterial contamination could be effectively reduced and completely removed after undergoing five different cleaning protocols (autoclave, chlorhexidine, chlorhexidine + saline, NaOCl solution, and NaOCl solution + saline). The present study tested decontamination methods that are readily available materials in the clinical setting, such as chlorhexidine, saline, and NaOCl solution, in addition to steam, autoclaving, and ultrasonic bath. This was decided upon in order to suggest simple and non-time-consuming protocols that can be effective and easily adopted by clinicians.

Direct and indirect biological reactions have been discussed in the literature related to the presence of micro-contamination on the transmucosal abutment area and basal implant–abutment connection. Contaminated surfaces can not only influence the process of initial soft tissue healing and attachment negatively but also provoke an inflammatory hard tissue reaction with increased osteoclast activity [18,39]. The results of an in vitro study, according to Canullo et al. [27], confirmed the presence of pollutants on the abutment surface, connection, and screw following traditional milling procedures, even after cleaning with steam. The presence of contaminants at the platform–abutment level has been addressed as a possible cause for different direct and indirect biologic and biomechanical responses. As a direct response, it must be noted that at the early stages of the peri-implant tissue healing process, contaminants might negatively influence the interactions between cellular components and materials, as these interactions are mediated by the state of the surface [39]. Additionally, Mishra et al. [18] longitudinally described that metallic micro-pollutants could be associated with tissue-damaging inflammation, with consequent osteoclastogenesis. As an indirect response, it can be supposed that microbiological factors (enhanced plaque accumulation due to micro-pollutants) may longitudinally affect the stability of peri-implant tissues. In fact, contamination is recognized to affect the titanium oxide layer [40], and according to Teughels et al. [41], it enhances the roughness of the abutment surface.

Furthermore, analyzing contamination from a mechanical point of view, such debris and oxide layers may affect the mechanical stability of the implant–abutment connection, increasing the implant–abutment microgap [42]. In fact, despite tolerance between the implant and the abutment, the presence of contaminants on the connection and screw could negatively affect preloading and thus mechanical stability. Although the longitudinal impact of pollutants has not yet been clinically demonstrated—except by a preclinical short-term study [43]—cleaning protocols could supposedly enhance implant–prosthesis integration.

The results of the present in vitro examination are supported by recent investigations. Canullo et al. demonstrated micro-contamination of various origins in in vitro studies on prefabricated and customized milled titanium abutments [21,27]. In the present study, these contaminants were significantly reduced due to the cleaning procedures applied.

It is important to note the limitations of this study:

The investigation is an in vitro study in a controlled environment with minimal confounding variables. The mouth is a much more complicated environment. In order to validate the results of this research, in vivo studies need to be conducted.

In addition, although the study investigated a broad number of mechanical and chemical decontamination methods, other methods such as plasma of argon were not included in this study. Furthermore, the study was conducted solely on titanium abutments, whereas other types of abutment materials, such as zirconia, are used in the dental field. Future studies on these topics are encouraged.

## 5. Conclusions

The different decontamination methods showed that putting the abutments in chlorhexidine resulted in significantly higher toxicity of the abutments to the surrounding cells, while all other samples showed values that were similar to the control. Steam cleaning seemed to be the most efficient method for reducing areas of debris and metallic contamination. The results of the mean bacterial counts showed that steam cleaning and ultrasonic bath treatment did not lower the bacterial load, while the other five decontamination methods significantly reduced contamination, with mean bacterial counts of 0.

## Figures and Tables

**Table 1:** XTT assay descriptive results. One-way ANOVA was used to assess the significance of the differences between the various groups.

Group	Mean OD	Std. Deviation	Std. Error
Autoclave n = 3	3.63	0.15	0.09
Ultrasonic n = 4	3.41	0.37	0.19
Steam n = 3	3.29	0.22	0.12
CHX n = 5	0.70	0.30	0.13
NaOCl n = 2	3.54	0.09	0.06
Prep non-decont. n = 3	3.48	0.23	0.13
Factory n = 3	3.62	0.04	0.02
F df = 6, 16	78.896 *		

* The mean difference is significant at the 0.05 level.

**Table 2:** Multiple comparisons (Scheffe’s test). Dependent variables—XTT results.

Group (I)	Group (J)	Mean Difference (I–J)	Std. Error	Sig.
CHX	Autoclave	−2.93	0.19	<0.001
	Ultrasonic	−2.71	0.17	
	Steam	−2.59	0.19	
	NaOCl	−2.84	0.21	
	Prep non-decont.	−2.78	0.19	
	Factory	−2.92	0.19	

**Table 3:** Mean bacterial count (CFU/mL) following the decontamination procedures of the tested samples. Bacterial growth was observed on the abutments treated with steam cleaning and ultrasonic bath, while very low growth was measured on the abutments treated with NaOCl solution. No bacterial growth was observed on the implant abutments treated with autoclave, chlorhexidine, chlorhexidine + saline, and NaOCl solution + saline. One-way ANOVA was used to assess the significance of the differences among the various groups.

Group	Mean (CFU/mL)	Std. Deviation
Steam	3.18 × 10^9^	2.36 × 10^8^
Autoclave	0	0
Ultrasonic	1.85 × 10^9^	1.29 × 10^8^
CHX	0	0
NaOCl	1 × 10^8^	1.15 × 10^8^
CHX + S	0	0
NaOCl + S	0	0
Control Unadjusted untreated abutments	2.75 × 10^9^	1.91 × 10^8^
Control Adjusted untreated abutments	2.83 × 10^9^	2.06 × 10^8^
